# Ulcerative colitis immune cell landscapes and differentially expressed gene signatures determine novel regulators and predict clinical response to biologic therapy

**DOI:** 10.1038/s41598-021-88489-w

**Published:** 2021-04-27

**Authors:** Harrison M. Penrose, Rida Iftikhar, Morgan E. Collins, Eman Toraih, Emmanuelle Ruiz, Nathan Ungerleider, Hani Nakhoul, Erik F. Flemington, Emad Kandil, Shamita B. Shah, Suzana D. Savkovic

**Affiliations:** 1 Department of Pathology and Laboratory Medicine, Tulane University, 1430 Tulane Ave SL-79, New Orleans, LA 70112 USA; 2 Division of Endocrine and Oncologic Surgery, Department of Surgery, Tulane University, New Orleans, LA 70112 USA; 3 Division of Gastroenterology, Ochsner Clinic Foundation, New Orleans, LA 70121 USA

**Keywords:** Inflammation, Computational biology and bioinformatics, Immunology, Gastroenterology

## Abstract

The heterogeneous pathobiology underlying Ulcerative Colitis (UC) is not fully understood. Using publicly available transcriptomes from adult UC patients, we identified the immune cell landscape, molecular pathways, and differentially expressed genes (DEGs) across patient cohorts and their association with treatment outcomes. The global immune cell landscape of UC tissue included increased neutrophils, T CD4 memory activated cells, active dendritic cells (DC), and M0 macrophages, as well as reduced trends in T CD8, Tregs, B memory, resting DC, and M2 macrophages. Pathway analysis of DEGs across UC cohorts demonstrated activated bacterial, inflammatory, growth, and cellular signaling. We identified a specific transcriptional signature of one hundred DEGs (UC_100_) that distinctly separated UC inflamed from uninflamed transcriptomes. Several UC_100_ DEGs, with unidentified roles in UC, were validated in primary tissue. Additionally, non-responders to anti-TNFα and anti-α4β7 therapy displayed distinct profiles of immune cells and pathways pertaining to inflammation, growth, and metabolism. We identified twenty resistant DEGs in UC non-responders to both therapies of which four had significant predictive power to treatment outcome. We demonstrated the global immune landscape and pathways in UC tissue, highlighting a unique UC signature across cohorts and a UC resistant signature with predictive performance to biologic therapy outcome.

## Introduction

Inflammatory bowel disease (IBD) includes two major inflammatory disorders known as Crohn’s Disease (CD) and Ulcerative Colitis (UC), and has a complex pathogenesis linked to genetic predisposition, microbial imbalances, elevated intestinal permeability, and a dysregulated immune response^1–3^. In intestinal tissue, epithelial and immune cells communicate to maintain homeostasis and their aberrant composition and interactions are critical in initiating and driving IBD pathobiology^2,4–6^. An early sign of intestinal inflammation is an elevated level of neutrophils that further fuels IBD progression, in part, by exacerbating tissue damage and through production of inflammatory cytokines^6,7^. Aberrant subsets of T cells and macrophages in the intestine are critical for facilitating inflammatory responses and injury in IBD pathobiology^4,5,8,9^. Some immune cells have dual functions, for example increased B cells and dendritic cells (DC) initially are protective, but in the long-term can contribute to IBD progression^10–12^. Moreover, in IBD tissue, the release of cytokines and chemokines by epithelial and immune cells can worsen disease^6,13^. These mediators, together with gut bacteria, facilitate activation of epithelial and immune cellular pathways including Toll-like receptors, TNFα receptors, NFκB, and JAK-STAT further exacerbating intestinal inflammation^2,5^. Blockade of several of these pathways including TNFα have provided plausible treatment options for certain IBD patients^5,14^.

Substantial progress has been made in defining the roles of individual immune cells and molecular pathways driving IBD^6,15^, yet the underlying pathobiology of disease heterogenicity and (non)response to therapy is not well understood. Moreover, distinctions between pediatric and adult IBD, as well as UC (colon) and CD (entire intestine), creates additional challenges for utilizing similar diagnostic and treatment options. Thus, to further expand understanding of disease our study focused on adult UC cohorts to identify shared immune cell landscapes and pathways in affected colon across patients, and to determine differences that may impact outcome to therapy. Here, we used publicly available transcriptomes of colonic tissue from a large number of adult UC patients to identify global immune landscapes, molecular pathways, and DEGs across cohorts. We identified a UC transcriptional signature that differentiates inflamed colonic tissue from matched uninflamed controls. The significance of this signature was validated in an independent cohort and several transcripts with unidentified roles in UC were further validated by qPCR. Moreover, we recognized immune cells, pathways, and DEGs of UC patients lacking response to biologic therapy and defined a resistant transcriptional signature with significant predictive power for non-responsiveness to therapy.

## Results

### A global immune cell landscape in healthy and UC tissue

**Figure 1:**
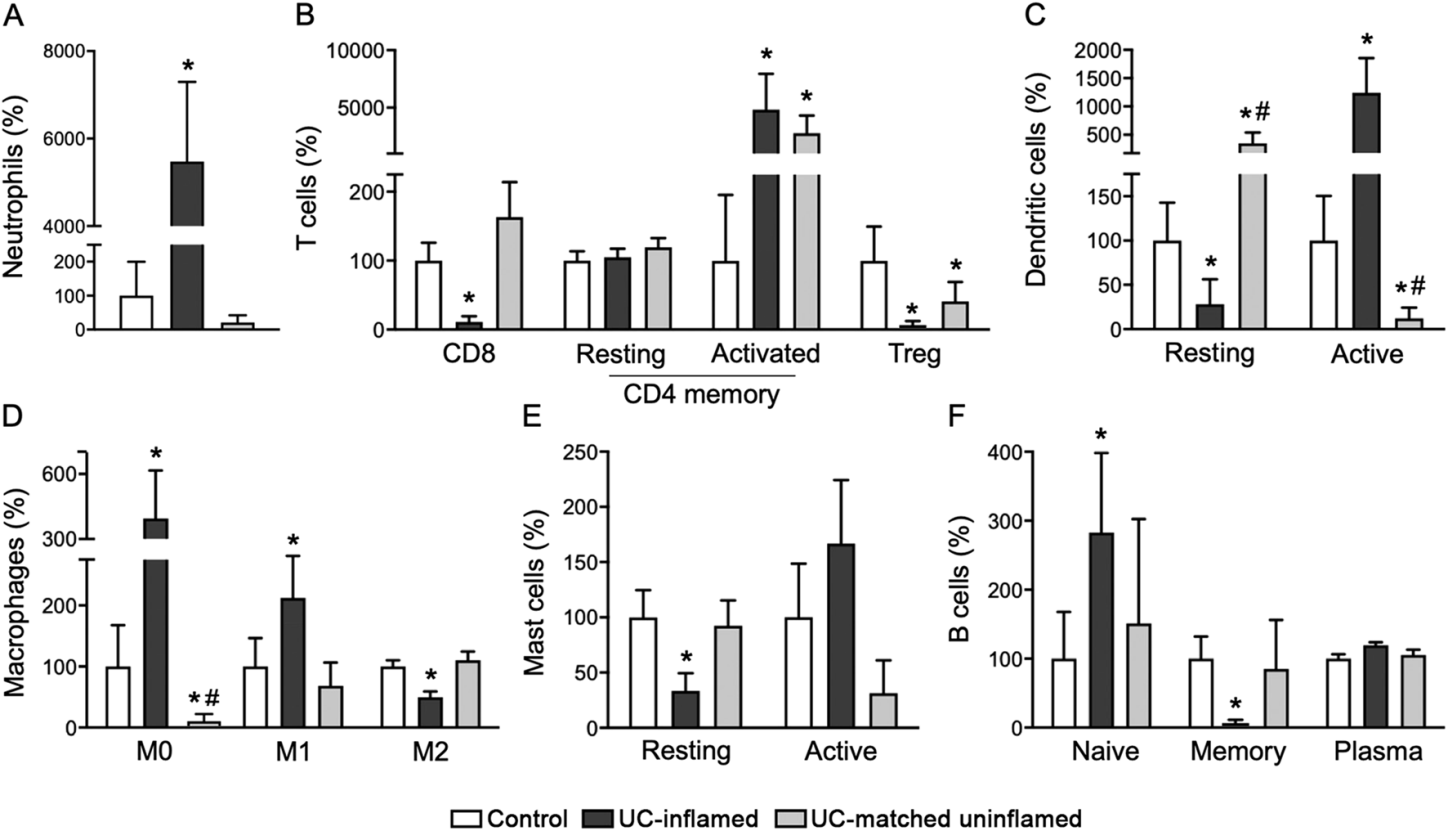
Alterations in immune cell profiles in UC inflamed and uninflamed matched tissue transcriptomes. CIBERSORT assessment of immune cell transcriptional signatures in transcriptomes of UC patients from inflamed or matched uninflamed tissue compared to healthy control. Immune cell signatures represent neutrophils (**A**) subsets of T cells (**B**), DC (**C**), macrophages (**D**), mast cells (**E**) and B cells (**F**) (GSE38713) (n = 13 healthy control, n = 15 UC-inflamed, n = 7 UC-matched uninflamed; CIBERSORT, *^#^*p* < 0.05; *vs. Con, ^#^vs. UC-inflamed).

We determined the global immune cell landscape of healthy and adult UC colonic tissue by assessing publicly available transcriptomes for abundances of immune cell transcriptional signatures using CIBERSORT^16,17^. The immune cell population of healthy colonic tissue consisted primarily of B cells (plasma), T cells (CD4 memory resting, Tregs), and macrophages (M2) across multiple groups (Supplementary Fig. S1). UC tissue revealed an altered immune cell presence compared to healthy controls (Supplementary Fig. S1). Specifically, these changes (Fig. 1A–F) included substantially elevated levels of neutrophils (50-fold, *p* < 0.05), T CD4 memory activated cells (50-fold, *p* < 0.05), active DC (12-fold, *p* < 0.05), M0/M1 macrophages (threefold, *p* < 0.05), and B naïve cells (threefold, *p* < 0.05) in UC tissue relative to healthy control. Several cell subsets showed reduced trends such as T CD8 cells, Tregs, B memory cells (by 99%, *p* < 0.05), and M2 macrophages (by 50% *p* < 0.05). Both resting DC and resting mast cells were lowered in UC tissue (*p* < 0.05) while their active forms showed elevated trends. Relative abundances of other cell subsets in UC tissue were also altered but did not meet significant thresholds with applied CIBERSORT criteria. These findings were graphically presented from a cohort (GSE38713) that included healthy controls, inflamed UC, and matched uninflamed tissues (Fig. 1A–F). A comparable immune cell landscape showing similar trends (neutrophils, DC, macrophages, mast cells, B cells, and subsets of T cells) was seen in four additional UC cohorts (GSE4183, GSE9452, GSE14580, GSE59071) with the exception of T CD4 resting cells and eosinophils (Supplemental Fig. S1). Moreover, in uninflamed (matched) colonic tissue from patients with active UC, we found the majority of the samples displayed an immune cell landscape similar to healthy colonic tissue. They also shared some similarities with inflamed UC as demonstrated by increased CD4 memory activated and reduced Tregs cells (Fig. 1B, *p* < 0.05). The presence of DC (resting and active) and macrophages (M0) differed from both healthy and inflamed UC tissue (Fig. 1C,D, *p* < 0.05).

### Remodeled molecular pathways and a transcriptional signature for inflamed UC tissue

**Figure 2:**
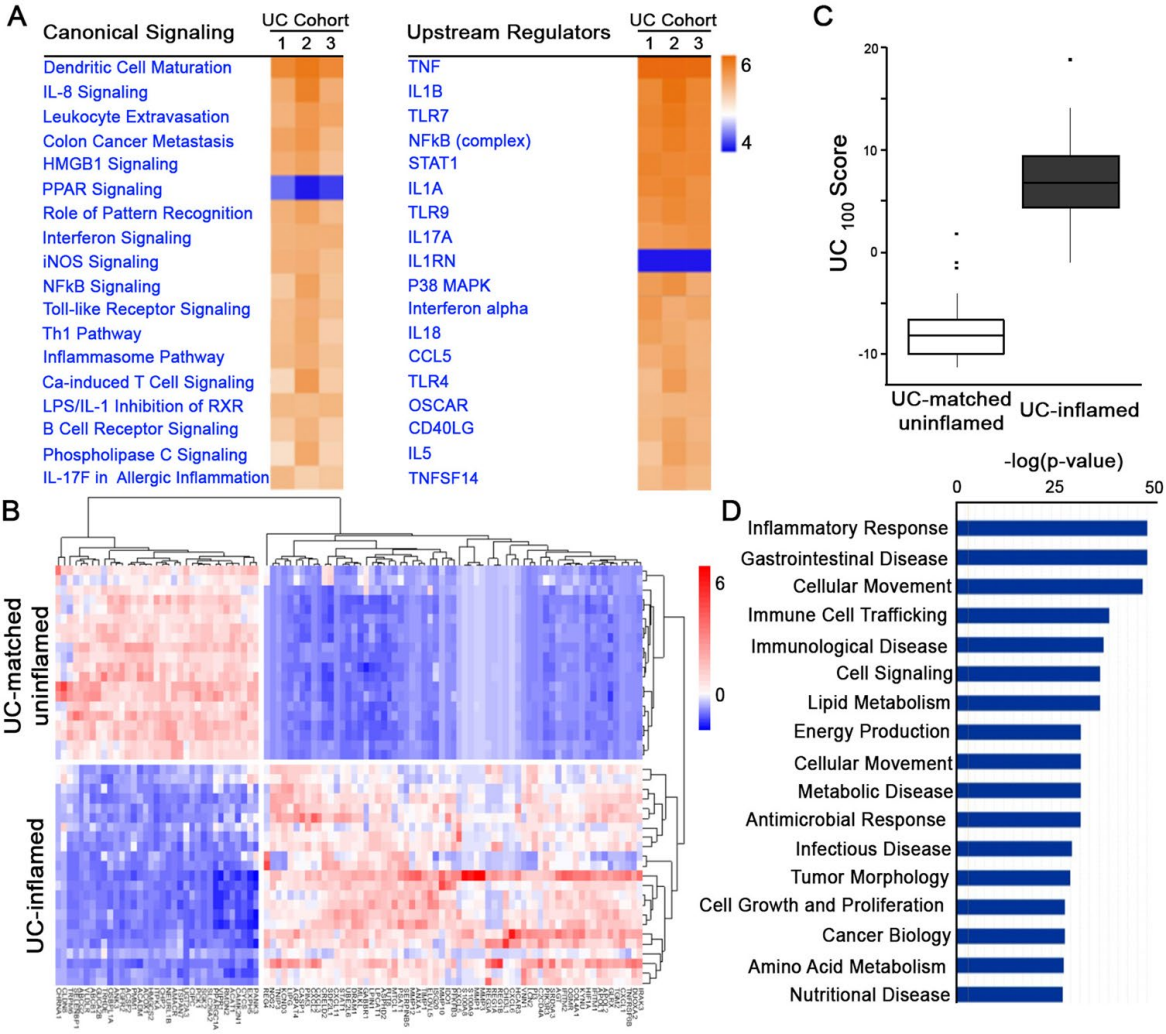
Altered molecular pathways in inflamed UC tissue. (**A**) IPA canonical pathway and upstream regulator analysis revealed similarly enriched pathways across three independent cohorts (1: GSE4183, n = 8 healthy control, n = 9 UC-inflamed; 2: GSE14580, n = 6 healthy control, n = 24 UC-inflamed; 3: GSE38713, n = 13 healthy control, n = 15 UC-inflamed; IPA, FDR < 0.05). (**B**) Hierarchical clustering, as shown by representative heatmap, revealed two distinct clusters of UC samples separated by the UC_100_ signature (generated from GSE4183, GSE14580, GSE38713) differentiating between inflamed UC and matched uninflamed transcriptomes from an independent cohort (GSE107593) (n = 24 UC-inflamed, n = 24 UC-matched uninflamed). (**C**) Presence of the UC_100_ signature represented as a score in inflamed UC compared to uninflamed matched control transcriptomes from an independent cohort (GSE107593) (*p* = 4.65e^-08^). (**D**) Disease and function enrichment analysis for UC_100_ signature (IPA, *p* < 0.05).

We determined remodeled molecular pathways associated with differentially expressed genes (DEGs) in UC tissue compared to controls using three cohorts (GSE4183, GSE38713, GSE14580) (Fig. 2A) (IPA, FDR < 0.05). Similarly activated pathways and upstream regulators were linked to bacterial response (TLRs, LPS/IL1), inflammation (Th1/2 responses, inflammasome: IL18, chemokine/cytokine activation: TNFα, IL-8, IFN, IL1, IL17), and intracellular signaling (NFκB, p38/MAPK). Further, we identified a panel of DEGs consistent across the same three cohorts (GSE4183, GSE38713, GSE14580) relative to controls (fold-change > |2| and adjusted *p* < 0.001). The top 100 DEGs (UC_100_) included 65 increased and 35 decreased relative to control (Supplementary Table S1) and were used for unsupervised clustering of an independent UC cohort (GSE107593). The UC_100_ distinctly separated inflamed from uninflamed samples in this independent cohort (Fig. 2B,C).

**Figure 3:**
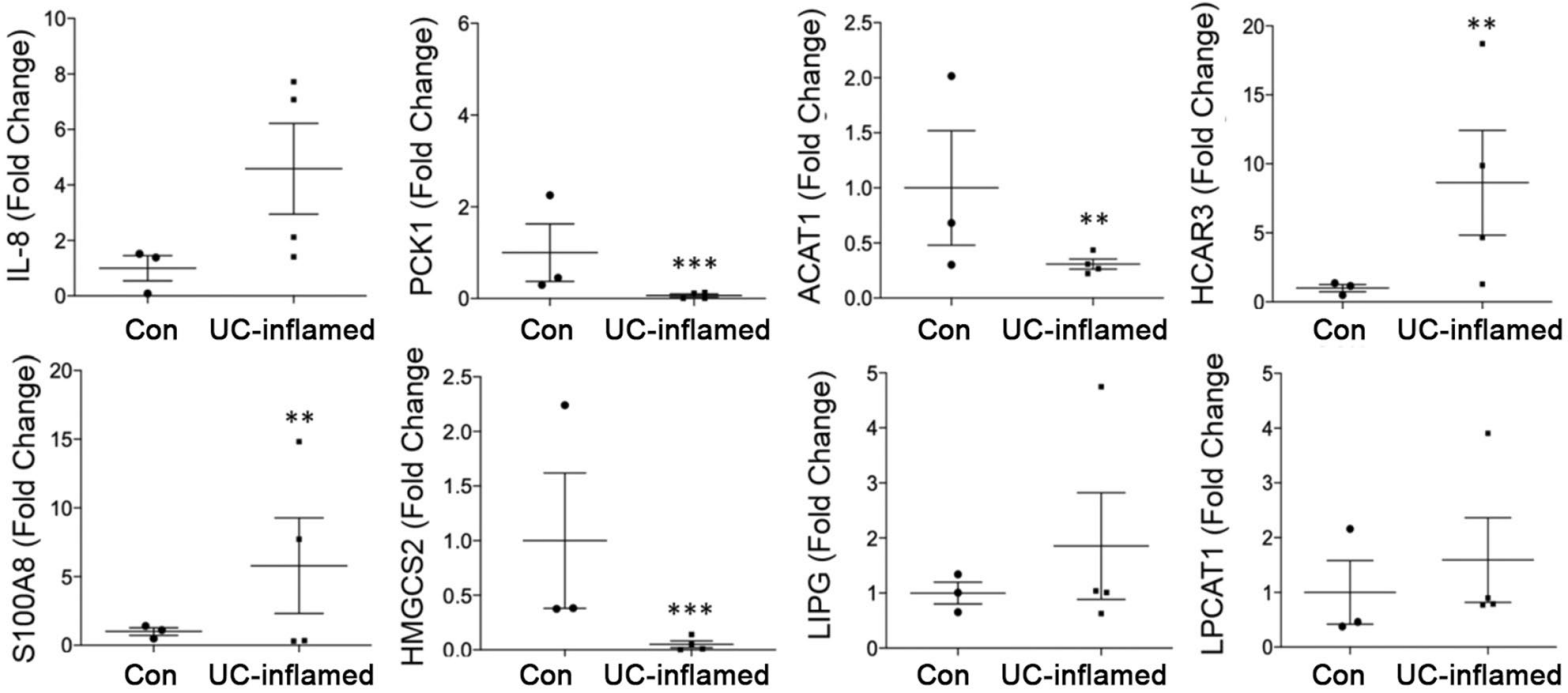
Altered expression of UC_100_ transcripts in UC tissue. Altered levels of select, transcripts from the UC_100_ signature (IL8, S100A8, PCK1, HMGCS2, ACAT1, HCAR3, LIPG, LPCAT1) in inflamed UC tissue relative to healthy control was confirmed by qPCR (control n = 3, UC n = 4, **p < 0.01, ***p < 0.001).

Collectively, DEGs from the UC_100_ signature encoded protein involved in multiple cellular functions such as bacterial response, inflammatory response, immune cell trafficking, growth, cell signaling as well as metabolic processing and lipid metabolism (Fig. 2D). Several of them encoded protein with established roles in UC pathobiology such as hypoxia (HIF1A), nitric oxide (NOS2), inflammation (TNIP3, TNFRSF6B, CXCL, IL1RN, IRAK3, IRF1, IFITM1, OMSR), matrix metallopeptidases (MMP1, 3, 10, 12), and calcium signaling (S100A8)^6,18–21^. There were also DEGs whose roles in UC pathobiology have not been explored, which we validated by qPCR (Fig. 3). We confirmed altered expression of established IL8 and S100A8 and novel transcripts PCK1 (Phosphoenolpyruvate Carboxykinase 1), HMGCS2 (3-Hydroxy-3-Methylglutaryl-CoA Synthase 2), ACAT1 (Acetyl-CoA Acetyltransferase 1), HCAR3 (Hydroxycarboxylic Acid Receptor 3), LPCAT1 (Lysophosphatidylcholine Acyltransferase 1), and LIPG (Lipase G, Endothelial Type). These novel DEGs encode protein functionally involved in metabolism of lipids, glucose, and mitochondria suggesting that metabolic responses related to glucose metabolism and mitochondrial function were attenuated (decreased PCK1, HMGCS2, ACAT1) while lipid metabolism/signaling was elevated (increased HCAR3, LIPG, LPCAT1) (total n = 7, *p < 0.05).

### Distinct immune cell landscapes, pathways, and DEGs in UC tissue lacking response to biologic treatments

**Figure 4:**
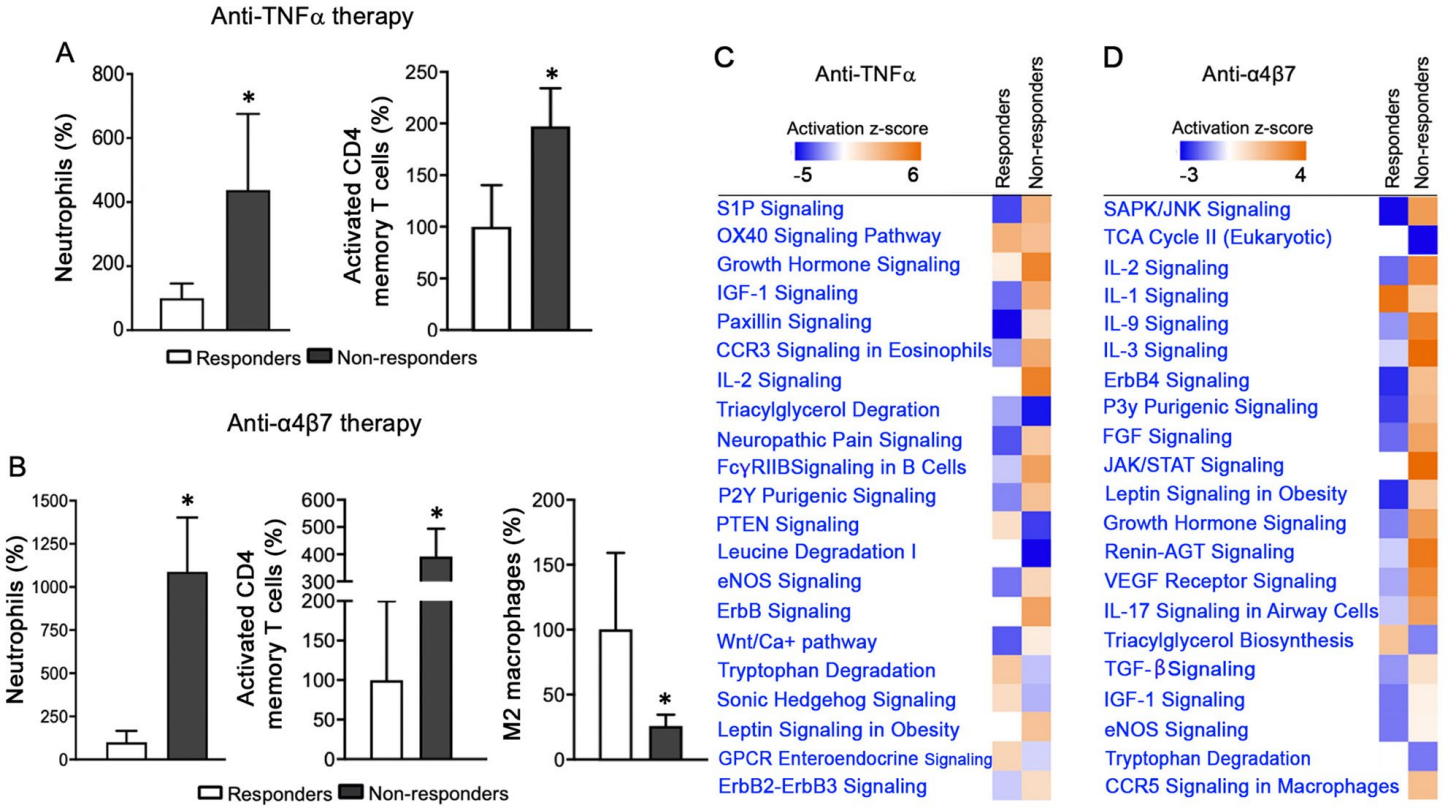
Immune cell landscapes and molecular pathways in non-responders prior to anti-TNFα and anti-α4β7 therapy. (**A**, **B**) CIBERSORT of UC tissue transcriptomes from non-responders prior to therapy showed an increased abundance in neutrophils, activated T CD4 memory, and reduced macrophages M2 compared to responders (GSE73661) (n = 8 anti-TNFα responders, n = 15 anti-TNFα non-responders; n = 9 anti-α4β7 responders, n = 25 anti-α4β7 non-responders CIBERSORT, *p < 0.05). (**C**, **D**) IPA canonical pathway analysis revealed enriched molecular pathways in non-responders to anti-TNFα and anti-α4β7 (GSE73661) (IPA, DEGs responders vs. control; non-responders vs. control, FDR < 0.05).

We further determined whether immune cell landscapes, molecular pathways, and DEGs were distinct to UC tissue of patients non-responsive to biologic anti-TNFα and anti-α4β7 therapy. We utilized publicly available transcriptomes of UC tissue acquired from patients (GSE73661) before anti-TNFα and anti-α4β7 treatments that were later classified as non-responders or responders by clinical endoscopic assessment for disease remission status^22,23^. CIBERSORT revealed that UC tissue of non-responders relative to responders, prior to anti-TNFα and anti-α4β7 therapy, had considerably increased neutrophils (4 to 10-fold, *p* < 0.05) (Fig. 4A,B) and T CD4 activated cells (2 to 4-fold, *p* < 0.05) (Fig. 4A,B). Further, those non-responsive to anti-α4β7 treatment showed reduced levels of M2 macrophages (by more than 50%, *p* < 0.05) (Fig. 4B). Additionally, we analyzed pathways of UC non-responders (vs. control) relative to UC responders (vs. control) before anti-TNFα and anti-α4β7 treatments. Non-responders demonstrated activation of distinct molecular pathways linked to aberrant immune responses (IL1-3, IL17, CCR3), growth (VEGF, TGF, IGF1, Wnt/Ca2+, Erb2/3/4, growth hormone), and energy metabolism (leptin, sphingolipase, triglyceride degradation, TCA cycle) relative to responders (Fig. 4C,D) (IPA, FDR < 0.05).

**Figure 5:**
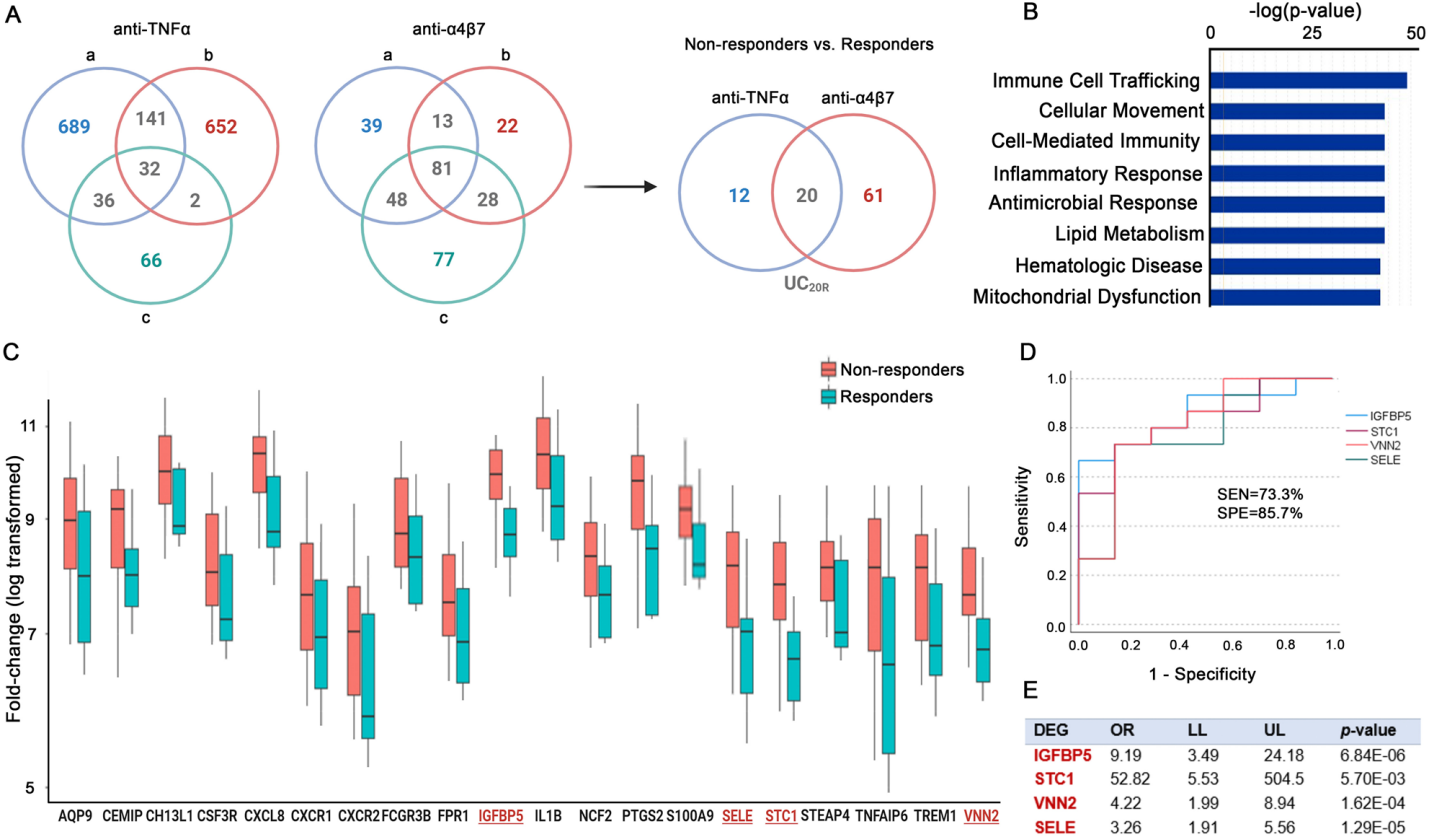
Differentially expressed genes in non-responders to anti-TNFα and anti-α4β7 treatment reflecting treatment “failure” and “prediction” to therapy outcomes. (**A**) DEGs were generated from samples (GSE12251, GSE73661) obtained during and after anti-TNFα treatment (non-responders vs. responders) following different dosage and time points (a: 5 mg/kg (8 weeks; GSE12251), b: 10 mg/kg (8 weeks; GSE12251), c: 10 mg/kg (4–6 weeks; GSE73661). Additional DEGs were generated from samples obtained during and after the course of anti-α4β7 therapy (a: 6 weeks, b: 12 weeks, c: 52 weeks; GSE73661). Intersection between resistant gene following anti-TNFα (32 genes) and anti-α4β7 (81 genes) yielded 20 shared DEGs representing “failure” to both therapies, i.e. resistant DEGs (UC_20R_) (LIMMA R Package, *p* < 0.05, FC |1.5|, DEGs). (**B**) Canonical pathway analysis revealed the top enriched molecular pathways in the UC_20R_ panel from non-responders to anti-TNFα and anti-α4β7 (IPA, FDR < 0.05). (**C**) Expression levels of the UC_20R_ genes in independent UC samples obtained before anti-TNFα and anti-α4β7 treatment of UC patients (GSE73661). Box plot represents the fold-change (log transformed) of selected DEGs in prospective non-responders (NR) and responders (R). Significantly upregulated IGFBP5, SELE, STC1, and VNN2 were marked in red font. (**D**) “Predictive” performance of the IGFBP5, SELE, STC1, and VNN2 panel, as determined by ROC curve analysis and calculating AUC in both anti-TNFα and anti-α4β7 non-responders before therapy (*p* < 0.05). Combined gene analysis of pretreatment data of UC patients (GSE73661) was employed and resulting sensitivity and specificity. (**E**) Multivariate regression analysis showed overexpression of the four genes was associated with higher risk of treatment failure. *OR* odds ratio, *LL* lower limit of 95% confidence interval, *UL* upper limit of 95% confidence interval.

Moreover, we determined whether differences in DEGs between non-responders and responders could reveal treatment “failure” and identified those with “predictive” power for treatment outcome. Initial analysis of transcriptomes from UC patients (GSE12251, GSE73661) obtained during and after anti-TNFα and anti-α4β7 treatments identified DEGs in non-responders vs. responders representing “failure” to the treatment (Fig. 5A). Specifically, we recognized DEGs in non-responders that were resistant to therapy (“failure”), among which 32 were found in the anti-TNFα group (30 upregulated and 2 downregulated) and 81 from the anti-α4β7 group (68 upregulated and 13 downregulated) (fold-change > |1.5| and adjusted *p* < 0.05) (Fig. 5A) (Supplementary Tables S2 and S3). Further, among these resistant DEGs we found 20 (UC_20R_) represented “failure” for both treatments (Fig. 5A) (Table 1). These resistant UC_20R_ encoded protein associated with response to bacteria, defense response, cell surface receptors, cell signaling, cell trafficking, endothelial function, lipid metabolism, and mitochondrial dysfunction (Fig. 5B) (IPA, FDR < 0.05). Next, we assessed their “predictive” significance in independent samples obtained prior to treatment (GSE73661). Their expression levels in UC pretreatment datasets showed significant differences in prospective non-responders relative to responder groups (Fig. 5C, fold-change |1.5|, *p* < 0.05). Specifically, we found the top four significantly increased transcripts, IGFBP5 (Insulin Like Growth Factor Binding Protein 5), SELE (Selectin E), STC1 (Stanniocalcin 1), and VNN2 (Vanin 2), in non-responders. Moreover, IGFBP5, SELE, STC1, and VNN2 had significant “predictive” power for determining (non)response to both anti-TNFα and anti-α4β7 as demonstrated by receiver operating characteristic (ROC) curve analysis and calculating area under the curve (AUC) (*p < 0.05) (Fig. 5D) (Table 2). Further, multivariate regression analysis showed elevated levels of these four transcripts were associated with higher risk of treatment failure (Fig. 5E).

**Table 1:** UC_20R_ resistance gene signature representing the top 20 differentially expressed genes from UC tissue of patients non-responsive to both biologic anti-TNFα and anti-α4β7 compared to responders. (GSE73661) (GSE12251) (n = 25 non-responders, 21 responders for anti-TNFα; n = 35 non-responders, 18 responders for anti-α4β7).

Gene	Gene name	Anti-TNFα		Anti-α4β7	
		Fold-change	adj.P.Val	Fold-change	adj.P.Val
CHI3L1	Chitinase 3 like 1	2.6	2.7e − 04	3.8	8.9E − 11
AQP9	Aquaporin 9	3.0	8.3e − 05	3.0	3.1E − 07
CXCL8	Chemokine ligand 8	2.9	2.7e − 05	2.8	2.0E − 07
SELE	Selectin E	2.5	1.8e − 04	2.7	7.1E − 07
CEMIP	Cell migration inducing hyaluronan binding protein	2.1	3.1e − 07	2.3	8.9E − 07
IL1B	Interleukin 1 beta	1.9	1.4e − 04	2.3	5.9E − 06
TNFAIP6	TNF alpha induced protein 6	2.3	5.4e − 04	2.3	4.9E − 05
STEAP4	STEAP4 metalloreductase	1.6	4.4e − 04	2.3	4.3E − 07
FCGR3B/FCGR3A	Fc fragement of IgG receptor IIIB/Fc fragment of IgG IIIa	2.3	1.9e − 05	2.3	1.9E − 07
CXCR1	Chemokine receptor 1	3.1	8.3e − 05	2.2	2.9E − 06
IGFBP5	Insulin like growth factor binding protein 5	1.7	1.7e − 03	2.1	2.4E − 07
PTGS2	Prostaglandin-endoperoxide synthase 2	2.4	2.0e − 04	2.1	4.9E − 06
CSF3R	Colony stimulating factor 3 receptor	2.0	2.3e − 04	2.1	1.3E − 06
S100A9	S100 calcium binding protein A9	2.2	2.3e − 04	2.0	3.4E − 06
STC1	Stanniocalcin 1	2.0	2.8e − 06	1.9	3.4E − 09
VNN2	Vanin 2	2.4	1.0e − 04	1.9	2.6E − 05
TREM1	Triggering receptor expressed on myeloid cells 1	2.5	2.0e − 04	1.9	2.6E − 05
NCF2	Neutrophil cytosolic factor 2	2.2	6.2e − 05	1.7	8.0E − 05
CXCR2	Chemokine receptor 2	2.2	2.1e − 04	1.7	8.0E − 05
FPR1	Formyl peptide receptor 1	1.8	8.1e − 05	1.6	3.5E − 05

**Table 2:** Predictive performance of UC_20R_ resistance gene signature for treatment response to anti-TNFα and anti-α4β7. (GSE73661, GSE12251) (n = 25 non-responders, 21 responders for anti-TNFα; n = 35 non-responders, 18 responders for anti-α4β7).

Gene	Anti-TNFα			Anti-α4β7		
	AUC	SD	p value	AUC	SD	p value
AQP9	0.93	0.06	0.001	0.89	0.04	< 0.001
CEMIP	0.88	0.07	0.003	0.92	0.05	< 0.001
CHI3L1	0.92	0.06	0.001	0.96	0.02	< 0.001
CSF3R	0.98	0.03	< 0.001	0.93	0.05	< 0.001
CXCL8	0.98	0.03	< 0.001	0.93	0.04	< 0.001
CXCR1	0.89	0.07	0.002	0.92	0.04	< 0.001
CXCR2	0.89	0.07	0.002	0.96	0.04	< 0.001
FCGR3B/3A	0.98	0.03	< 0.001	0.91	0.02	< 0.001
FPR1	0.96	0.04	< 0.001	0.93	0.04	< 0.001
IGFBP5	0.84	0.08	0.008	0.96	0.04	< 0.001
IL1B	0.93	0.05	< 0.001	0.93	0.03	< 0.001
NCF2	0.94	0.05	< 0.001	0.88	0.05	< 0.001
PTGS2	0.91	0.06	0.002	0.90	0.05	< 0.001
S100A9	0.93	0.06	< 0.001	0.97	0.05	< 0.001
SELE	0.93	0.06	< 0.001	0.89	0.02	< 0.001
STC1	1.00	0.00	0.001	0.91	0.05	< 0.001
STEAP4	0.88	0.08	0.003	0.89	0.04	< 0.001
TNFAIP6	0.92	0.06	0.001	0.85	0.04	< 0.001
TREM1	0.91	0.07	0.002	0.91	0.05	< 0.001
VNN2	0.93	0.06	0.001	0.92	0.04	< 0.001

## Discussion

We demonstrated in adult UC patients, global immune landscapes and molecular pathways in affected colonic tissue and identified those distinct to non-responders to biologic therapy. Further, we identified a transcriptional signature common across UC cohorts and a resistant signature specific for patients non-responsive to biologic therapy. We validated altered expression of several novel DEGs whose roles in UC pathobiology are unexplored. These findings provide insight into new genes with altered expression in UC tissue that could serve as potential biomarkers for precise diagnostics and targets for personalized therapeutic interventions for UC patients.

We found strong similarities in immune landscapes among UC cohorts represented by altered neutrophils, DC, macrophages, mast cells, B cells, and subsets of T cells which are recognized to have key roles in IBD pathogenesis^4–6^. Further, in UC tissue we showed elevated M1 and reduced M2 populations, which have pro- and anti-inflammatory roles. Depending on their polarization, macrophages are able to foster each other’s activity, increase activation of DC, and communicate with adaptive T and B cells in promoting inflammation^5,24–26^. T CD8 cell levels could vary in UC affected tissue due to variation in their subsets, and their plasticity may be attributed to dynamic interplay between intestinal tissue and circulating cells^27,28^. Further, decreased trends in T CD8 cells may result in weak antigen presentation and processing by intestinal epithelial cells^29^. Moreover, we found that abundances of the T CD4 memory activated subset differed between cohorts. Aberrant T CD4 responses lead to poor defense to pathogens and has been observed to vary among IBD patients, which in combination with disbalances in gut microbiota could be responsible for disease relapse^9,30^. Moreover, T CD4 and Tregs cells are found in uninflamed matched tissue from UC patients with active disease. When Tregs are dysregulated or deficient, the intestine is one of the first tissues that becomes inflamed due to constant immune stimulation by microbiota and food antigens^9,31^. In active IBD, Tregs can expand in the lamina propria, but their immuno-suppressive activity is diminished^32,33^. We speculate that differences in these subsets of T cells between patient cohorts could also be due to the composition of gut microbiota and regional diet. Similarly, variation in eosinophils, which play roles in protecting barrier integrity and immunity might be related to geographic and seasonal disparities among UC cohorts^34,35^. We observed increased trends in activated mast cells in UC tissue compared to control. Mast cell levels could vary in UC affected tissue depending on location of affected colon and inflammation status, and may account for variability that we and others have observed^36^. Recent findings revealed the presence of activated DCs and plasmacytoid DCs in colonic biopsies of UC and CD patients using the xCell platform^37^. Furthermore, single-cell sequencing data from one UC cohort^38^ provides classification of multiple subsets of epithelial and stromal cells including inflammatory fibroblast, monocyte, microfold, and T cell networks^38^. Further development of new approaches differentiating active vs. non-active immune cells and interactive vs. non-interactive cells may provide for more precision-based identifiers of cell landscapes in IBD tissue.

We identified similarly altered molecular pathways and DEGs in UC tissue across cohorts. These pathways are linked to bacterial and inflammatory signaling. Further, the UC_100_ signature distinguished inflamed from uninflamed transcriptomes. UC_100_ DEGs encode protein with established roles in UC inflammation, hypoxia, nitric oxide and matrix metallopeptidases^6,18–21^ as well as those with novel, unexplored roles in IBD pathobiology. Many of these DEGs encoded protein functionally linked to metabolic energy functions such as alterations in lipid, glucose, and mitochondrial functions. Emerging findings show that aberrant energy metabolism may become another hallmark of IBD. Elevated lipids can drive intestinal inflammation, and in mouse models, blockade of their production ameliorates inflammation^39–42^. Newly identified DEGs from the UC_100_ signature increased in UC tissue, LIPG and LPCAT1, are regulators of lipid metabolism. LPCAT1 encodes an enzyme responsible for the conversion of lysophosphatidylcholine to phosphatidylcholine and is involved in the regulation of lipid droplet number and size^43^. Limited studies showed that increased lipid droplets may drive intestinal inflammation^40,44^ and LPCAT1 could play an important function in the synthesis of inflammatory lipids^45^. LIPG is a member of the triglyceride lipase family and may be involved in lipoprotein metabolism and endothelial biology^46^. Further, HCAR3 is involved in regulation of lipolysis during increased β-oxidation and may play integral roles in crosstalk between microbiome-derived metabolites and immune cells^47,48^. PCK1, decreased in UC, is a regulator of gluconeogenesis and its deficiency in macrophages was demonstrated to facilitate a proinflammatory phenotype^49^. Moreover, HMGCS2 and ACAT1, both decreased in UC, encode regulators of mitochondrial function and both play important roles in β-oxidation. In intestinal stem cells, HMGCS2 has a vital role in regulation of cellular differentiation and homeostasis, and its loss could impact barrier renewal and function^50,51^. ACAT1 plays an important role in ketone body metabolism and recently was implicated in inflammatory responses in macrophages as well as diet-induced obesity^52^. Another important aspect of altered energy dynamics in intestinal inflammation involves mitochondrial function. In IBD intestine, mitochondrial gene expression is aberrant leading to reduced respiratory activity and energy depletion, associated with bacterial signaling^53–57^. Smillie et al. suggested that metabolic alterations in intestinal cells and monocytes represented by a shift from oxidative phosphorylation to glycolysis may be driven by impaired production of microbiota short-chain fatty acids leading to upregulated pathways for dietary fatty acids^38,58^. Furthermore, a recent study described mitochondrial fission–fusion as critical in driving dysregulation of intestinal cells and macrophages, which could be targeted as a possible therapeutic approach^59^. Additionally, it is important to consider the effects of environmental factors in mediating mitochondrial reprograming such as the use of antibiotics and intake of a high-fat western style diet^60^. While the exact mechanisms and roles of metabolic reprograming in intestinal cells and immune cells are not fully understood, their emergence as a hallmark of intestinal inflammation highlights their critical importance in the underlying pathobiology of disease.

In UC patients non-responsive to anti-TNFα or anti-α4β7 therapy, we identified distinct immune cell landscapes, molecular pathways, and transcriptional signatures relative to responders. Increased abundances of neutrophils and activated subsets of T cells in the colon of UC non-responders to both treatments suggest that severity of disease might be a predictive factor for success to biologic therapy. Clinical studies demonstrated that non-responsiveness to the biologic therapy is, in part, related to disease severity, a patient’s age at diagnosis, and duration of inflammation^61–64^. Furthermore, in UC patients non-responsive to anti-α4β7 treatment, we observed reduced M2 macrophage levels. Verstockt et al. transcriptomic analysis of UC tissue from patients prior to receiving biologic therapy revealed significant enrichment of immune cells in non-responders including M1 macrophages and Tregs, while responders had elevated naïve B cells^65^. Our CIBERSORT analysis also showed a trend in elevated M1 macrophages, while changes in Tregs were insignificant. Another report described enrichment of monocytes, M1 macrophages, activated DCs and plasmacytoid DC subsets in non-responders to biologic therapy^37^. While our approach did not provide significant increases in monocyte or M1 macrophage levels, they trended up in non-responder groups. Moreover, non-responders to biologic therapy have displayed higher inflammatory markers and cytokines in circulating monocytes^66^. Thus, based on several of these cellular characteristics in non-responders, further development of immune profile-based signatures could allow for precise diagnostics and optimal therapy selection in the future. Moreover, DEGs and molecular pathways are distinct for non-responders to anti-TNFα or anti-α4β7 therapy, highlighted by biologic functions pertaining to inflammation, growth, lipid metabolism, and mitochondrial dysfunction. We determined a specific resistance UC_20R_ signature representing “failure” to biologic therapy. Among them the top four differentially expressed genes, STC1, VNN2, SELE, and IGFBP5, possess significant “predictive” power to both anti-TNFα or anti-α4β7 therapy. SELE, VNN2, and STC1 play critical roles in neutrophil accumulation and transendothelial movement at sites of inflammation, suggesting a possible role for immune cell trafficking in non-responders^67^. Moreover, these novel and distinct features of disease could also occur, in part, because of changes in the microbiota caused by therapy^68^. Thus, we anticipate that transcriptional signatures found in UC patient tissue may guide selection of therapy and more personalized therapeutic approaches. With further advances in these technologies, we will expand understanding of systemic and specific changes in immune profiles, pathways, and transcripts to include other aspects of IBD heterogeneity including those from adult UC and CD as well as pediatric patients.

Here, with a comprehensive assessment of UC colonic tissue, we demonstrated both shared and distinct immune cell landscapes and molecular pathways. Our results could provide insight into disease pathogenesis and mechanistic reasons why certain patients do not respond to mainstay therapy. Utilization of bioinformatics approaches in combination with human genetics, epigenetics, and single-cell genomics will lead to better understanding of inflammatory disorders, risk of disease recurrence, and association with treatment outcomes leading to development of more precise, personalized diagnostics and therapeutic intervention for adult and pediatric IBD.

## Materials and methods

### Data sources of ulcerative colitis patient transcriptomes

Ulcerative colitis (UC) colonic tissue microarray and RNAseq gene expression datasets used in this study were obtained from the National Center for Biotechnology Information Gene Expression Omnibus (NCBI GEO) data repository using the following GEO accession numbers listed in Table 3. In total, 326 adult patient transcriptomes were analyzed from European and American cohorts^22,23,69–75^; pediatric UC patients were excluded from analysis. For healthy control (n = 42), we analyzed colonic transcriptomes obtained from individuals undergoing colonoscopy for either moderate gastrointestinal symptoms or colon cancer screening. Transcriptomes from UC patients included regions of inflamed (n = 154) and matched uninflamed control (n = 31). Additionally, we utilized transcriptomes from UC patients prior to and during anti-TNFα (infliximab) (n = 46) and anti-α4β7 integrin (vedolizumab) (n = 53) treatment (Table 3)^22,23^.

**Table 3:** Clinical and demographic characteristics of UC patients included in analysis.

Cohort accession	Platform	Total	Control	UC	Gender (%F)	Median age
GSE9452	Microarray (Affymetrix HG-U133_Plus_2)	13	5^a^	8	28%	46
GSE38713	Microarray (Affymetrix HG-U133_Plus_2)	35	20^a^^,b^	15	45%	42
GSE14580	Microarray ((Affymetrix HG-U133_Plus_2)	30	6^a^	24	41%	43
GSE59071	Microarray (Affymetrix HuGene-1_0-st)	84	10^a^	74	41%	45
GSE4183	Microarray (Affymetrix HG-U133_Plus_2)	17	8^a^	9	66%	N/A
GSE107593	RNAseq (Illumina NextSeq 500)	48	24^b^	24	N/A	N/A

Cohort accession	Platform	Total	Responder	Non-responder	Gender (%F)	Median age
GSE12251 (TNF)	Microarray (Affymetrix HG-U133_Plus_2)	23	13	10	58%	37
GSE73661 (TNF)	Microarray (Affymetrix HuGene-1_0-st)	23	8	15	43%	41
GSE73661 (VDZ)	Microarray (Affymetrix HuGene-1_0-st)	53	18	35	48%	40

^a^Healthy control.

^b^Uninflamed matched control from UC patients.

### Differential expression testing and pathway analysis

Differentially expressed genes (DEGs) from UC microarray datasets (GSE4183, GSE14580, GSE38713) were identified using the web-based NCBI GEO2R software application^76^. Only those genes expressed at a minimum threshold of > |2.0|-fold change as compared to healthy control (with an adjusted *p* < 0.05) were used for pathway analysis. Ingenuity Pathway Analysis (IPA) (www.qiagen.com/ingenuity) was used to generate canonical pathway and disease and function analyses in the Tulane Cancer Center Next Generation Sequence Analysis Core (www.tulane.edu/som/cancer/research/core-facilities/cancer-crusaders). IPA analysis included appropriate use of background genes through pre-analysis filtering of ‘species filtering’ (i.e. human) as well ‘tissue and cell lines’ to include those relevant to the intestine (i.e. intestinal cells, immune cells, stromal cells, and others).

### Microarray processing and generation of a multi-cohort UC transcriptional signature and UC biologic resistance signature

We generated a single list of DEGs by combining microarray data from three independent UC cohorts (GSE4183, GSE38713, GSE14580). Only cohorts from the same microarray platform (Affymetrix Human Genome U133 Plus 2.0 Array platform) were utilized in an effort to reduce for inconsistencies among probe identifiers and batch effects across different samples were controlled for through SVA^77^. Transcriptomes were initially managed and normalized using the multi-array average method (justRMA) from the affy Bioconductor package (v. 1.60.0)^78^. Differential expression testing between inflamed UC tissue and healthy control samples was accomplished using the limma package (v. 3.38.2)^79,80^. Any genes duplicated in analysis were filtered and only those genes meeting an adjusted *p* < 0.05 were used. Transcripts were selected based on the most significant statistical significance and fold-change > |2.0| differences. Generation of biologic resistance signatures were accomplished using two microarray datasets from UC patients treated with anti-TNFα (GSE12251, GSE73661) or anti-α4β7 (GSE73661). DEGs were identified in non-responders compared to responders at various time points using the limma R differential expression analysis package and mean calculation was performed for gene-level summarization. The Benjamini & Hochberg test was used to estimate the adjusted p value (*p* < 0.05).

### RNAseq processing

Processing of RNAseq was accomplished in the Tulane Cancer Center Next Generation Sequence Analysis Core (www.tulane.edu/som/cancer/research/core-facilities/cancer-crusaders). Raw RNAseq reads (GSE107593) were mapped to an index containing the human haploid genome sequence (Genome Reference Consortium *Homo sapiens* genome build 38, GRCh38). For quantification of RNAseq data the software program, RSEM (v.1.2.25)^81^ was employed. Analysis of transcript reads as measured by Fragments Per Kilobase of transcript per Million mapped reads (FPKM) were used further analysis.

### Hierarchical clustering

Hierarchical clustering of inflamed UC tissue and matched uninflamed control RNAseq transcriptomes (GSE107593) was achieved utilizing Cluster3 software using an uncentered correlation as a symmetric matrix, complete linkage, and Pearson correlation as the similarity measure. JavaTree software was used to create the corresponding heatmaps^82,83^.

### Principal Component Analysis (PCA) and generation of a transcriptional signature score

PCA of inflamed UC tissue and matched uninflamed control RNAseq transcriptomes (GSE107593) was accomplished using the FactoMineR R package and PCA function^84^. The first two coordinates of samples and their percent variation were plotted. For summarizing transcriptional expression of the multicohort signature into a single value, the following formulation was utilized:

$$Score = mean \left[ {log\;2\left( {\frac{x + 1}{m}} \right)} \right]$$

with *x* representing the expression value of the transcript and *m* representing the median of the transcripts similar to the approach previously used by Agrawal et al.^85^.

### CIBERSORT (cell type identification by estimating relative subsets of known RNA transcripts)

UC microarray gene expression datasets were formatted into mixture files with patient identifiers and corresponding gene expression levels; these files were subsequently uploaded for CIBERSORT analysis according to formatting requirements (http://cibersort.stanford.edu)^17^. Findings were further validated using updated CIBERSORTx analysis^16^. Analysis of mixture files was performed using the core LM22 signature consisting of 547 genes that precisely differentiate mature human hematopoietic cells to determine relative abundances of 22 immune cell subsets including: B-cells (naïve, memory, plasma cells), T-cells (CD8, naïve CD4, memory CD4, follicular helper, regulatory, γδ), monocytes, macrophages (M0, M1, M2), dendritic cells, mast cells, eosinophils, and neutrophils. Duplicated genes were filtered based on those meeting an adjusted *p* < 0.05 before being input into analysis. For those genes with multiple probes meeting significance thresholds, the average expression value of the probe identifiers was calculated and used for analysis. Immune cell output was reported as relative fractions for all immune cell subsets and represented as stacked bar charts as a proportion of one hundred percent or as fold-change differences normalized to healthy control or therapy responders.

### Primary UC tissue RNA sources

Colonic mucosal tissue biopsies for validation of transcript expression were obtained from actively inflamed tissue sections of patients with UC (Origene Technologies; ID: CR561752, CR562039, CR562979, CR561525, CR560265) and from normal colonic mucosal biopsies of patients who underwent colon cancer screening or tissue removal (ID: CR560498, CR560940, CR560136).

### qPCR

Total RNA obtained from human colonic tissue was utilized for cDNA preparation required for qPCR as previously described^56,86^. The primers used for amplification of human cDNA included: ACAT1 (F: 5′-GCCATTGAAAAGGCAGGGATT-3′; R: 5′-TGCCTTGTAGGAGCTTGTCC-3′), HMGCS2 (F: 5′-TACCACCAATGCCTGCTACG-3′; R: 5′-TGGCATAACGACCATCCCAG-3′), LPCAT1 (F: 5′-ATCCCGATCTGGGGAACTCT-3′; R: 5′-ATCTGTGGCCACTTTCCGTT-3′), HCAR3 (F: 5′-ATCTGGGCCCAACCTCAAAT-3′; R: 5′-TCTTAGGCCGAGTCCAGTGA-3′), LIPG (F: 5′-TGGTTTGAACGTGGGGAACT-3′; R: 5′-GTGTCAGTTTGAGGGTCTGCT-3′), PCK1 (F: 5′-CTGAACCTCTCGGCCAAAGT-3′; R: 5′-GAGAGCCAACCAGCAGTTGT-3′), IL8 (F: 5′-GTGCAGTTTTGCCAAGGAGT-3′; R: 5′-CTCTGCACCCAGTTTTCCTT-3′), S100A8 (F: 5′-TCAGCCCTGCATGTCTCTTC-3′; R: 5′-CGTCTGCACCCTTTTTCCTGA-3′), ACTIN (F: 5′-CATCGAGCACGGCATCGTCA-3′; R: 5′-TAGCACAGCCTGGATAGCAAC-3′). To determine the relative levels of mRNA the comparative Cq method was employed using Actin as a housekeeping control.

### Statistical analysis

Only those values meeting a significant threshold (*p* < 0.05) as determined by the CIBERSORT and GEO2R algorithms were included in analysis. Statistical analysis was performed between groups by Student’s (paired or unpaired) t-test, analysis of variance (ANOVA) test, and Student Newman-Keuls post-test using Graph Pad Instat 3 software (Graph Pad Software). The prognostic performance of differentially expressed genes for predicting outcomes to therapy was estimated by receiver operating characteristic curve analysis and the area under the curve (AUC). The prognostic performance of differentially expressed genes for predicting outcomes to therapy was estimated by receiver operating characteristic curve analysis and the area under the curve (AUC) with each gene plotted with a curve, and diagnostic accuracy measures (sensitivity and specificity) reflecting the value of the combined analysis.

## Supplementary Information

Supplementary Figure Legend.

Supplementary Figure S1.

Supplementary Table S1.

Supplementary Table S2.

Supplementary Table S3.
