# Supplementary material for: Ulcerative colitis immune cell landscapes and differentially expressed gene signatures determine novel regulators and predict clinical response to biologic therapy
Source: Sci Rep. 2021 Apr 27;11:9010. doi: 10.1038/s41598-021-88489-w (PMC8079702; doi:10.1038/s41598-021-88489-w)
Supplement: Supplementary file 1 — Supplementary Figure Legend. [file 41598_2021_88489_MOESM1_ESM.docx]

**Supplemental Figure S1.** Transcriptome profiling of immune cell signatures in colonic samples from healthy individuals and UC patients from five independent cohorts. Graphs represent individual immune cell subsets out of total immune cell populations in colon and does not account for colonic epithelial or stromal cell fractions (GSE59071: n=10 healthy control, n=74 UC-inflamed; GSE38713: n=13 healthy control, n=15 UC-inflamed, n=7 UC-matched uninflamed; GSE9452: n=5 healthy control, n=8 UC-inflamed; GSE14580: n=6 healthy control, n=24 UC-inflamed; GSE4183: n=8 healthy control, n=9 UC-inflamed) (CIBERSORT, *p*<0.05).

ssss
