# Supplementary figures and images for: Ulcerative colitis immune cell landscapes and differentially expressed gene signatures determine novel regulators and predict clinical response to biologic therapy
Source: Sci Rep. 2021 Apr 27;11:9010. doi: 10.1038/s41598-021-88489-w (PMC8079702; doi:10.1038/s41598-021-88489-w)

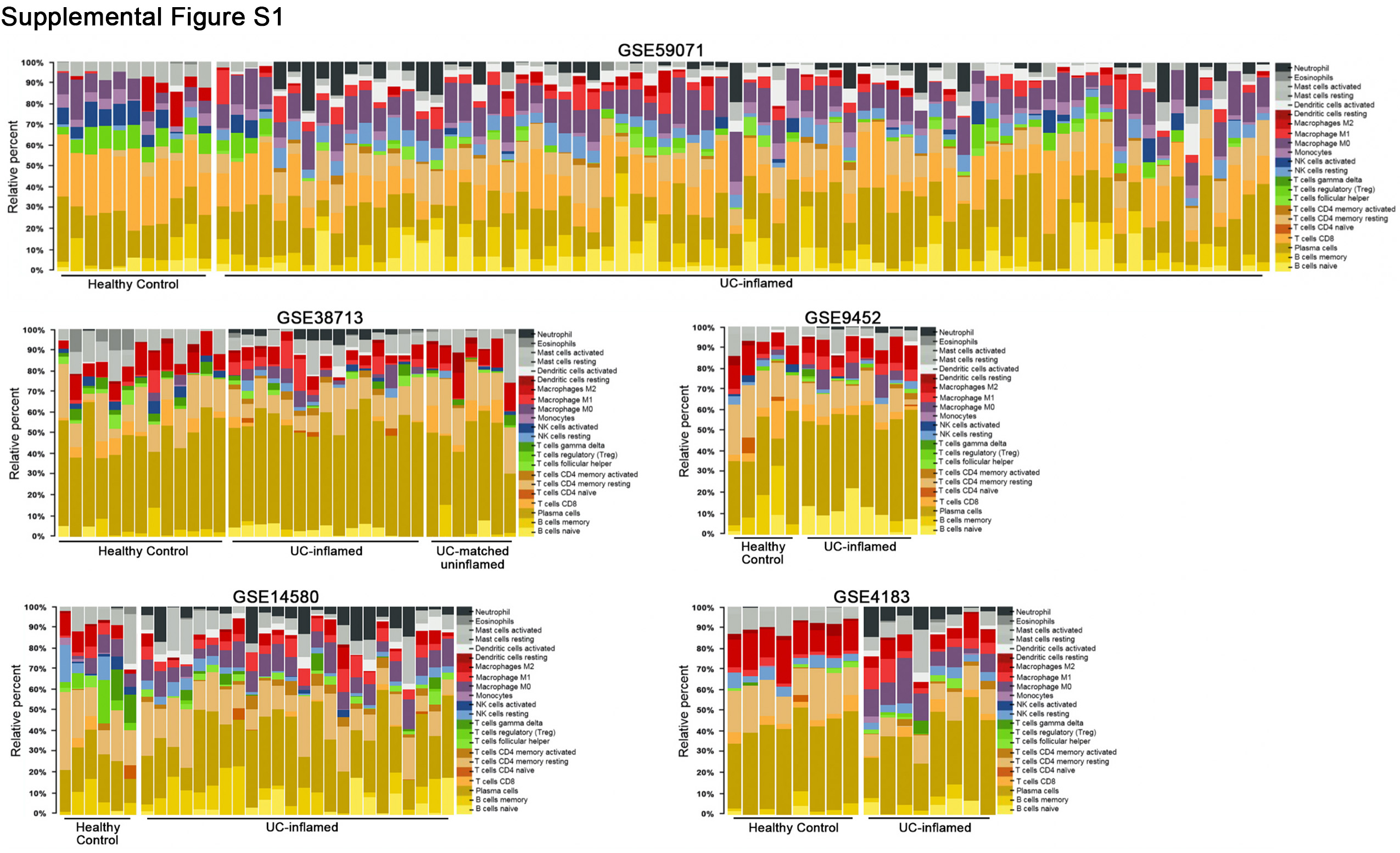

Supplement: Supplementary file 2 — Supplementary Figure S1. [file 41598_2021_88489_MOESM2_ESM.jpg]
