# Supplementary material for: Ulcerative colitis immune cell landscapes and differentially expressed gene signatures determine novel regulators and predict clinical response to biologic therapy
Source: Sci Rep. 2021 Apr 27;11:9010. doi: 10.1038/s41598-021-88489-w (PMC8079702; doi:10.1038/s41598-021-88489-w)
Supplement: Supplementary file 3 — Supplementary Table S1. [file 41598_2021_88489_MOESM3_ESM.docx]

**Supplementary Table S1:** UC_100_ signature representing the top 100 differentially expressed genes (DEGs) from UC colonic tissues relative to health control colon from a multi-cohort analysis. DEGs are presented by adj. p-value across combined cohorts. (n=27 healthy control, 48 UC-inflamed patients; GSE4183, GSE14580, GSE38713) (*potentially novel transcripts in UC pathobiology based on literature review).

| Gene | Gene name | Fold-change | adj.P.Val |  |
| --- | --- | --- | --- | --- |
| LPCAT1* | lysophosphatidylcholine acyltransferase 1 | 5.2 | 2.54E-34 |  |
| CXCL1 | C-X-C motif chemokine ligand 1 | 21.9 | 5.07E-32 |  |
| CHI3L1 | chitinase 3 like 1 | 34.0 | 7.12E-31 |  |
| MMP3 | matrix metallopeptidase 3 | 50.7 | 1.77E-28 |  |
| S100A8 | S100 calcium binding protein A8 | 50.3 | 2.09E-28 |  |
| DUOXA2 | dual oxidase maturation factor 2 | 14.2 | 3.91E-28 |  |
| HMGCS2* | 3-hydroxy-3-methylglutaryl-CoA synthase 2 | -6.9 | 5.40E-28 |  |
| KYNU* | Kynureninase | 16.6 | 5.40E-28 |  |
| UBE2L6 | ubiquitin conjugating enzyme E2 L6 | 3.1 | 6.82E-28 |  |
| KCND3* | potassium voltage-gated channel subfam D member 3 | 5.7 | 1.23E-27 |  |
| CRELD2 | cysteine rich with EGF like domains 2 | 2.6 | 2.01E-27 |  |
| LPIN1* | lipin 1 | 2.5 | 6.03E-26 |  |
| TIMP1 | TIMP metallopeptidase inhibitor 1 | 6.6 | 8.27E-26 |  |
| LCN2 | lipocalin 2 | 13.7 | 2.13E-25 |  |
| TGM2 | transglutaminase 2 | 5.1 | 4.07E-25 |  |
| IFITM2 | interferon induced transmembrane protein 2 | 3.3 | 2.22E-24 |  |
| REG1A | regenerating family member 1 alpha | 62.1 | 3.32E-24 |  |
| ACAT1* | acetyl-CoA acetyltransferase 1 | -2.8 | 3.55E-24 |  |
| CXCL6 | C-X-C motif chemokine ligand 6 | 16.9 | 1.19E-23 |  |
| ACSF2* | acyl-CoA synthetase family member 2 | -3.4 | 1.45E-23 |  |
| PI3 | peptidase inhibitor 3 | 11.7 | 5.46E-23 |  |
| MMP10 | matrix metallopeptidase 10 | 16.2 | 5.80E-23 |  |
| MMP12 | matrix metallopeptidase 12 | 9.9 | 6.64E-23 |  |
| AMACR | alpha-methylacyl-CoA racemase | -4.5 | 9.98E-23 |  |
| TRPM6 | transient receptor cation channel subfamily M member6 | -5.3 | 1.78E-22 |  |
| ABCB1 | ATP binding cassette subfamily B member 1 | -6.7 | 2.50E-22 |  |
| IFITM1 | interferon induced transmembrane protein 1 | 2.7 | 2.56E-22 |  |
| CASP1 | caspase 1 | 3.3 | 3.00E-22 |  |
| ANXA1 | annexin A1 | 7.7 | 4.15E-22 |  |
| ACOX1* | acyl-CoA oxidase 1 | -2.8 | 5.32E-22 |  |
| TNFRSF6B | TNF receptor superfamily member 6b | 4.4 | 5.68E-22 |  |
| CXCL3 | C-X-C motif chemokine ligand 3 | 12.3 | 6.71E-22 |  |
| SGK2* | SGK2, serine/threonine kinase 2 | -5.8 | 1.16E-21 |  |
| GABBR1* | gamma-aminobutyric acid type B receptor 1 | 10.0 | 1.41E-21 |  |
| LIPG* | lipase G, endothelial type | 3.7 | 1.62E-21 |  |
| REG4 | regenerating family member 4 | 10.4 | 2.12E-21 |  |
| MMP1 | matrix metallopeptidase 1 | 33.7 | 2.71E-21 |  |
| VLDLR* | very low density lipoprotein receptor | -4.9 | 3.17E-21 |  |
| STAT1 | signal transducer and activator of transcription 1 | 2.3 | 4.74E-21 |  |
| SLC26A2* | solute carrier family 26 member 2 | -12.0 | 5.36E-21 |  |
| CXCL5 | C-X-C motif chemokine ligand 5 | 35.3 | 5.63E-21 |  |
| TNIP3* | TNFAIP3 interacting protein 3 | 13.5 | 6.84E-21 |  |
| GLRX* | Glutaredoxin | 2.0 | 8.38E-21 |  |
| IRAK3 | interleukin 1 receptor associated kinase 3 | 4.1 | 1.33E-20 |  |
| PIK3R3 | phosphoinositide-3-kinase regulatory subunit 3 | 2.3 | 2.04E-20 |  |
| TSPAN7* | tetraspanin 7 | -3.5 | 4.33E-20 |  |
| NOS2 | nitric oxide synthase 2 | 4.8 | 4.70E-20 |  |
| C2CD4A* | C2 calcium dependent domain containing 4A | 8.1 | 4.71E-20 |  |
| FUT8 | fucosyltransferase 8 | 2.5 | 5.44E-20 |  |
| MLKL | mixed lineage kinase domain like | 2.3 | 5.45E-20 |  |
| PCK1* | phosphoenolpyruvate carboxykinase 1 | -14.6 | 1.09E-19 |  |
| IDO1 | indoleamine 2,3-dioxygenase 1 | 10.1 | 1.12E-19 |  |
| REG3A | regenerating family member 3 alpha | 41.2 | 1.23E-19 |  |
| CIPC* | CLOCK interacting pacemaker | -2.7 | 1.40E-19 |  |
| PIM3 | Pim-3 proto-oncogene, serine/threonine kinase | 2.3 | 2.46E-19 |  |
| CYCS* | cytochrome c, somatic | -2.4 | 2.89E-19 |  |
| DRAM1* | DNA damage regulated autophagy modulator 1 | 3.0 | 3.76E-19 |  |
| ELOVL5* | ELOVL fatty acid elongase 5 | 3.8 | 4.38E-19 |  |
| PFKFB3 | 6-phosphofructo-2-kinase/fructose-2,6-biphosphatase 3 | 5.0 | 6.38E-19 |  |
| RMDN2* | regulator of microtubule dynamics 2 | -3.4 | 6.43E-19 |  |
| PMM1* | phosphomannomutase 1 | -2.1 | 6.88E-19 |  |
| ABCG2* | ATP binding cassette subfamily G member 2 | -11.8 | 6.94E-19 |  |
| SERPINB5 | serpin family B member 5 | 10.9 | 7.08E-19 |  |
| ACADM | acyl-CoA dehydrogenase, C-4 to C-12 straight chain | -2.1 | 7.22E-19 |  |
| VNN1 | vanin 1 | 15.3 | 7.37E-19 |  |
| VIPR1 | vasoactive intestinal peptide receptor 1 | -3.1 | 8.51E-19 |  |
| REG1B | regenerating family member 1 beta | 51.3 | 8.87E-19 |  |
| CAMK2N1* | calcium/calmodulin dependent protein kinase inhibitor 1 | -2.5 | 9.87E-19 |  |
| OSMR | oncostatin M receptor | 4.5 | 5.41E-19 |  |
| SELENBP1 | selenium binding protein 1 | -4.3 | 1.17E-18 |  |
| AGT | Angiotensinogen | 3.1 | 1.17E-18 |  |
| CHRNA1 | cholinergic receptor nicotinic alpha 1 subunit | -2.5 | 1.36E-18 |  |
| ISG20* | interferon stimulated exonuclease gene 20 | 2.9 | 1.77E-18 |  |
| SDF2L1* | stromal cell derived factor 2 like 1 | 2.1 | 2.06E-18 |  |
| HIF1A | hypoxia inducible factor 1 alpha subunit | 2.2 | 2.17E-18 |  |
| ASPHD2* | aspartate beta-hydroxylase domain containing 2 | 3.1 | 2.61E-18 |  |
| S100A9 | S100 calcium binding protein A9 | 7.0 | 3.11E-18 |  |
| SRD5A3* | steroid 5 alpha-reductase 3 | 3.7 | 3.48E-18 |  |
| CHP2* | calcineurin like EF-hand protein 2 | -7.3 | 3.75E-18 |  |
| PPARGC1A* | PPARG coactivator 1 alpha | -3.3 | 4.14E-18 |  |
| ITPKA* | inositol-trisphosphate 3-kinase A | -2.1 | 4.40E-18 |  |
| MTCL1* | microtubule crosslinking factor 1 | 3.0 | 4.44E-18 |  |
| CLDN8 | claudin 8 | -24.1 | 5.47E-18 |  |
| APOL1* | apolipoprotein L1 | 3.3 | 7.19E-18 |  |
| PANK3* | pantothenate kinase 3 | -2.1 | 7.29E-18 |  |
| NEURL1B* | neuralized E3 ubiquitin protein ligase 1B | -3.4 | 7.66E-18 |  |
| OSBPL1A* | oxysterol binding protein like 1A | -2.8 | 7.75E-18 |  |
| GUCA2B | guanylate cyclase activator 2B | -8.3 | 8.66E-18 |  |
| TRHDE* | thyrotropin releasing hormone degrading enzyme | -5.0 | 1.07E-17 |  |
| COL4A1* | collagen type IV alpha 1 chain | 4.5 | 1.45E-17 |  |
| ANK3* | ankyrin 3, node of Ranvier (ankyrin G) | -3.3 | 1.56E-17 |  |
| AGPAT4* | 1-acylglycerol-3-phosphate O-acyltransferase 4 | 3.0 | 2.51E-17 |  |
| PSAT1* | phosphoserine aminotransferase 1 | 3.7 | 2.85E-17 |  |
| FGFR2 | fibroblast growth factor receptor 2 | -2.3 | 3.45E-17 |  |
| HCAR3* | hydroxycarboxylic acid receptor 3 | 12.1 | 4.52E-17 |  |
| UGT2A3* | UDP glucuronosyltransferase family 2 member A3 | -16.2 | 5.28E-17 |  |
| CXCL2 | C-X-C motif chemokine ligand 2 | 11.4 | 6.34E-17 |  |
| APOL2 | apolipoprotein L2 | 2.2 | 6.63E-17 |  |
| CXCL11 | C-X-C motif chemokine ligand 11 | 14.3 | 6.73E-17 |  |
| EXPH5* | exophilin 5 | -4.7 | 7.71E-17 |  |
