# Supplementary material for: Ulcerative colitis immune cell landscapes and differentially expressed gene signatures determine novel regulators and predict clinical response to biologic therapy
Source: Sci Rep. 2021 Apr 27;11:9010. doi: 10.1038/s41598-021-88489-w (PMC8079702; doi:10.1038/s41598-021-88489-w)
Supplement: Supplementary file 4 — Supplementary Table S2. [file 41598_2021_88489_MOESM4_ESM.docx]

**Supplementary Table S2:** Resistant gene signature representing top 32 differentially expressed genes from UC tissue of patients non-responsive to biologic anti-TNFα (GSE73661, GSE12251) (n=25 non-responders, 21 responders).

| Gene | Gene name | Fold-change | adj.P.Val |
| --- | --- | --- | --- |
| CXCR1 | C-X-C motif chemokine receptor 1 | 8.6 | 8.0E-05 |
| AQP9 | aquaporin 9 | 8.0 | 8.0E-05 |
| CXCL8 | C-X-C motif chemokine ligand 8 | 7.5 | 3.0E-05 |
| HCAR3 | hydroxycarboxylic acid receptor 3 | 6.5 | 7.0E-04 |
| CHI3L1 | chitinase like 1 | 6.1 | 3.0E-04 |
| TREM1 | triggering receptor expressed on myeloid cells 1 | 5.7 | 2.0E-04 |
| SELE | selectin E | 5.7 | 2.0E-04 |
| PTGS2 | prostaglandin-endoperoxide synthase 2 | 5.3 | 2.0E-04 |
| VNN2 | vanin 2 | 5.3 | 1.0E-04 |
| TNFAIP6 | TNF alpha induced protein 6 | 4.8 | 5.00E-04 |
| FCGR3B/  FCGR3A | Fc fragment of IgG receptor IIIb/Fc fragment of IgG receptor IIIa | 4.8 | 2.0E-05 |
| CXCR2 | C-X-C motif chemokine receptor 2 | 4.7 | 2.0E-04 |
| S100A9 | S100 calcium binding protein A9 | 4.5 | 2.0E-04 |
| CEMIP | cell migration inducing hyaluronan binding protein | 4.3 | 3.0E-04 |
| OSM | oncostatin M | 4.3 | 3.0E-04 |
| IL6 | interleukin 6 | 4.3 | 5.0E-04 |
| IL13RA2 | interleukin 13 receptor subunit alpha 2 | 4.1 | 7.0E-04 |
| STC1 | stanniocalcin 1 | 4.1 | 2.0E-04 |
| SRGN | serglycin | 4.0 | 1.0E-05 |
| CSF3R | colony stimulating factor 3 receptor | 3.9 | 3.0E-05 |
| IL1B | interleukin 1 beta | 3.8 | 1.0E-04 |
| MNDA | myeloid cell nuclear differentiation antigen | 3.7 | 1.0E-04 |
| FCGR1CP/  FCGR1B/  FCGR1A | Fc fragment of IgG receptor Ic, pseudogene/  Fc fragment of IgG receptor Ib/  Fc fragment of IgG receptor Ia | 3.7 | 9.0E-05 |
| NCF2 | neutrophil cytosolic factor 2 | 3.6 | 6.0E-05 |
| SELL | selectin L | 3.5 | 2.0E-03 |
| FPR1 | formyl peptide receptor 1 | 3.4 | 8.0E-05 |
| TAGAP | T-cell activation RhoGTPase activating protein | 3.2 | 2.0E-04 |
| GBP5 | guanylate binding protein 5 | 3.2 | 3.0E-04 |
| IGFBP5 | insulin like growth factor binding protein 5 | 3.2 | 2.0E-03 |
| STEAP4 | STEAP4 metalloreductase | 3.1 | 4.0E-04 |
| ADH1C | alcohol dehydrogenase 1C (class I), gamma polypeptide | -4.2 | 9.0E-04 |
| UGT2A3 | UDP glucuronosyltransferase family 2 member A3 | -4.3 | 2.0E-03 |
