# Supplementary material for: Ulcerative colitis immune cell landscapes and differentially expressed gene signatures determine novel regulators and predict clinical response to biologic therapy
Source: Sci Rep. 2021 Apr 27;11:9010. doi: 10.1038/s41598-021-88489-w (PMC8079702; doi:10.1038/s41598-021-88489-w)
Supplement: Supplementary file 5 — Supplementary Table S3. [file 41598_2021_88489_MOESM5_ESM.docx]

**Supplementary Table S3:** Resistant gene signature representing top 81 differentially expressed genes from UC tissue of patients non-responsive to biologic anti-α4β7 (GSE73661) (n=35 non-responders, 18 responders).

| Gene | Gene name | Fold-change | adj.P.Val |  |
| --- | --- | --- | --- | --- |
| S100A8 | S100 calcium binding protein A8 | 14.0 | 3E-12 |  |
| MMP3 | matrix metallopeptidase 3 | 13.8 | 2E-10 |  |
| CHI3L1 | chitinase 3 like 1 | 13.7 | 6E-15 |  |
| MMP7 | matrix metallopeptidase 7 | 9.1 | 1E-10 |  |
| MMP1 | matrix metallopeptidase 1 | 8.1 | 3E-08 |  |
| AQP9 | aquaporin 9 | 7.7 | 3E-09 |  |
| TCN1 | transcobalamin 1 | 7.6 | 1E-09 |  |
| TNIP3 | TNFAIP3 interacting protein 3 | 7.6 | 1E-11 |  |
| CXCL8 | C-X-C motif chemokine ligand 8 | 6.7 | 1E-09 |  |
| CXCL1 | C-X-C motif chemokine ligand 1 | 6.2 | 3E-15 |  |
| SELE | selectin E | 6.2 | 6E-09 |  |
| KYNU | kynureninase | 5.7 | 2E-13 |  |
| IDO1 | indoleamine 2,3-dioxygenase 1 | 5.6 | 6E-08 |  |
| OSMR | oncostatin M receptor | 5.0 | 2E-15 |  |
| SERPINA3 | serpin family A member 3 | 5.0 | 3E-13 |  |
| IL1B | interleukin 1 beta | 4.8 | 9E-08 |  |
| CEMIP | cell migration inducing hyaluronan binding protein | 4.8 | 1E-08 |  |
| RGS5 | regulator of G-protein signaling 5 | 4.6 | 6E-11 |  |
| SELP | selectin P | 4.5 | 9E-10 |  |
| STEAP4 | STEAP4 metalloreductase | 4.5 | 4E-09 |  |
| S100A12 | S100 calcium binding protein A12 | 4.5 | 2E-06 |  |
| TNC | tenascin C | 4.5 | 1E-07 |  |
| CXCR1 | C-X-C motif chemokine receptor 1 | 4.5 | 4E-08 |  |
| SPP1 | secreted phosphoprotein 1 | 4.4 | 1E-06 |  |
| TNFAIP6 | TNF alpha induced protein 6 | 4.4 | 4E-06 |  |
| FCGR2A | Fc fragment of IgG receptor IIa | 4.4 | 2E-07 |  |
| TDO2 | tryptophan 2,3-dioxygenase | 4.3 | 3E-06 |  |
| FCGR3B/  FCGR3A | Fc fragment of IgG receptor IIIb/  Fc fragment of IgG receptor IIIa | 4.2 | 2E-04 |  |
| PI15 | peptidase inhibitor 15 | 4.2 | 2E-04 |  |
| IGFBP5 | insulin like growth factor binding protein 5 | 4.2 | 2E-09 |  |
| MMP12 | matrix metallopeptidase 12 | 4.2 | 2E-08 |  |
| PTGS2 | prostaglandin-endoperoxide synthase 2 | 4.1 | 9E-08 |  |
| CSF3R | colony stimulating factor 3 receptor | 4.1 | 1E-08 |  |
| MGP | matrix Gla protein | 4.1 | 4E-09 |  |
| COL12A1 | collagen type XII alpha 1 chain | 4.0 | 2E-07 |  |
| ADGRL4 | adhesion G protein-coupled receptor L4 | 3.8 | 2E-09 |  |
| BGN | biglycan | 3.8 | 8E-10 |  |
| S100A9 | S100 calcium binding protein A9 | 3.8 | 7E-08 |  |
| SERPINE1 | serpin family E member 1 | 3.8 | 6E-09 |  |
| PLEK | pleckstrin | 3.7 | 3E-06 |  |
| IRAK3 | interleukin 1 receptor associated kinase 3 | 3.6 | 1E-16 |  |
| CALCRL | calcitonin receptor like receptor | 3.6 | 4E-09 |  |
| VWF | von Willebrand factor | 3.5 | 6E-11 |  |
| STC1 | stanniocalcin 1 | 3.5 | 4E-12 |  |
| VNN2 | vanin 2 | 3.5 | 2E-06 |  |
| TREM1 | triggering receptor expressed on myeloid cells 1 | 3.5 | 9E-07 |  |
| CXCL10 | C-X-C motif chemokine ligand 10 | 3.5 | 8E-06 |  |
| EDNRA | endothelin receptor type A | 3.4 | 4E-08 |  |
| ACSL4 | acyl-CoA synthetase long-chain family member 4 | 3.4 | 2E-08 |  |
| COL6A3 | collagen type VI alpha 3 chain | 3.3 | 3E-09 |  |
| NCF2 | neutrophil cytosolic factor 2 | 3.3 | 3E-08 |  |
| MMP9 | matrix metallopeptidase 9 | 3.3 | 6E-08 |  |
| CCDC3 | coiled-coil domain containing 3 | 3.2 | 3E-09 |  |
| COL4A1 | collagen type IV alpha 1 chain | 3.2 | 5E-10 |  |
| THY1 | Thy-1 cell surface antigen | 3.1 | 3E-08 |  |
| CXCR2 | C-X-C motif chemokine receptor 2 | 3.1 | 4E-06 |  |
| FAP | fibroblast activation protein alpha | 3.1 | 7E-06 |  |
| ALPL | alkaline phosphatase, liver/bone/kidney | 3.0 | 2E-08 |  |
| WNT5A | Wnt family member 5A | 3.0 | 3E-07 |  |
| FPR1 | formyl peptide receptor 1 | 3.0 | 2E-06 |  |
| ADGRF5 | adhesion G protein-coupled receptor F5 | 2.9 | 1E-09 |  |
| DYSF | dysferlin | 2.9 | 2E-08 |  |
| THBS2 | thrombospondin 2 | 2.9 | 3E-06 |  |
| KCNJ15 | potassium voltage-gated channel subfamily J member 15 | 2.9 | 2E-06 |  |
| G0S2 | G0/G1 switch 2 | 2.8 | 6E-06 |  |
| MME | membrane metallo-endopeptidase | 2.7 | 3E-06 |  |
| CXCL6 | C-X-C motif chemokine ligand 6 | 2.6 | 4E-06 |  |
| PECAM1 | platelet and endothelial cell adhesion molecule 1 | 2.4 | 2E-09 |  |
| EXPH5 | exophilin 5 | 3.4 | 1E-08 |  |
| CAPN13 | calpain 13 | 3.9 | 2E-13 |  |
| PADI2 | peptidyl arginine deiminase 2 | 4.1 | 2E-11 |  |
| UGT2A3 | UDP glucuronosyltransferase family 2 member A3 | 4.2 | 2E-13 |  |
| CWH43 | cell wall biogenesis 43 C-terminal homolog | 4.3 | 6E-08 |  |
| CDHR1 | cadherin related family member 1 | 4.9 | 1E-12 |  |
| ADH1C | alcohol dehydrogenase 1C (class I), gamma polypeptide | 5.3 | 1E-08 |  |
| GUCA2A | guanylate cyclase activator 2A | 5.6 | 2E-07 |  |
| CA1 | carbonic anhydrase 1 | 6.3 | 2E-06 |  |
| TMIGD1 | transmembrane and immunoglobulin domain containing 1 | 6.4 | 3E-08 |  |
| HMGCS2 | 3-hydroxy-3-methylglutaryl-CoA synthase 2 | 7.6 | 3E-11 |  |
| PCK1 | phosphoenolpyruvate carboxykinase 1 | 8.1 | 2E-11 |  |
| SLC26A2 | solute carrier family 26 member 2 | 9.0 | 2E-11 |  |
